# A Cardinal Role for Cathepsin D in Co-Ordinating the Host-Mediated Apoptosis of Macrophages and Killing of Pneumococci

**DOI:** 10.1371/journal.ppat.1001262

**Published:** 2011-01-27

**Authors:** Martin A. Bewley, Helen M. Marriott, Calogero Tulone, Sheila E. Francis, Timothy J. Mitchell, Robert C. Read, Benny Chain, Guido Kroemer, Moira K. B. Whyte, David H. Dockrell

**Affiliations:** 1 Medical School, University of Sheffield, Sheffield, United Kingdom; 2 Division of Infection and Immunity, University College London, London, United Kingdom; 3 Institute of Infection, Immunity and Inflammation, University of Glasgow, Glasgow, United Kingdom; 4 INSERM U848, Metabolomics Platform, Institut Gustave Roussy, Villejuif, France; 5 Centre de Recherche des Cordeliers, Paris, France; 6 Pôle de Biologie, Hôpital Européen Georges Pompidou, AP-HP, Paris, France; 7 Université Paris René Descartes, Paris, France; University of New Mexico, United States of America

## Abstract

The bactericidal function of macrophages against pneumococci is enhanced by their apoptotic demise, which is controlled by the anti-apoptotic protein Mcl-1. Here, we show that lysosomal membrane permeabilization (LMP) and cytosolic translocation of activated cathepsin D occur prior to activation of a mitochondrial pathway of macrophage apoptosis. Pharmacological inhibition or knockout of cathepsin D during pneumococcal infection blocked macrophage apoptosis. As a result of cathepsin D activation, Mcl-1 interacted with its ubiquitin ligase Mule and expression declined. Inhibition of cathepsin D had no effect on early bacterial killing but inhibited the late phase of apoptosis-associated killing of pneumococci *in vitro*. Mice bearing a cathepsin D^−/−^ hematopoietic system demonstrated reduced macrophage apoptosis *in vivo*, with decreased clearance of pneumococci and enhanced recruitment of neutrophils to control pulmonary infection. These findings establish an unexpected role for a cathepsin D-mediated lysosomal pathway of apoptosis in pulmonary host defense and underscore the importance of apoptosis-associated microbial killing to macrophage function.

## Author Summary

Tissue macrophages frequently undergo a program of cell death, termed apoptosis, following sustained ingestion and killing of bacteria. In macrophages, induction of apoptosis enhances bacterial killing when macrophages' initial killing capacity is exhausted. We have investigated the mechanism of apoptosis in macrophages exposed to pneumococci, the commonest cause of bacterial pneumonia. We show that the cell structure containing ingested bacteria, the phagolysosome, becomes permeabilized early in the death process. Pneumococcal exposure activates a phagolysosomal enzyme, cathepsin D, which induces apoptosis. Cathepsin D activation is required for permeabilization of mitochondria, an organelle implicated in apoptosis induction. Cathepsin D reduces levels of a negative regulator of apoptosis in macrophages, Mcl-1, by enhancing its association with an enzyme, which mediates its degradation. The importance of these findings was confirmed in a bone marrow transplant model in which mice either received bone marrow from mice containing or lacking the cathepsin D gene. This model showed that reduced apoptosis of alveolar macrophages occurred when cathepsin D was lacking, and that this impaired clearance of pneumococci in the mouse lung. We conclude that during bacterial challenge, lysosomal permeabilization and cathepsin D activation triggers a novel death pathway, in a timely fashion, linking bacterial killing to apoptosis induction.

## Introduction

Macrophages are essential for the maintenance of tissue homeostasis, as they remove dying and dead cells [1]. Macrophages must also coordinate the innate response to microorganisms that penetrate sterile environments such as the lower respiratory tract [2], [3]. To accommodate their opposing roles in long-term tissue homeostasis and short-term immune responses, tissue macrophages, such as alveolar macrophages, are long-lived in the basal state [4], [5], yet can activate a variety of death pathways upon pathogen encounter [6].

*Streptococcus pneumoniae*, the pneumococcus, is the most prevalent cause of community-acquired pneumonia [7]. During the initial stages of pneumococcal infection, macrophages are largely responsible for bacterial clearance and determine the initiation as well as the later resolution of the inflammatory response [8], [9]. Macrophage function is regulated by induction of apoptosis during pneumococcal infection [8], [10]. The shift from apoptosis resistance is determined by the decline in abundance of the anti-apoptotic protein Mcl-1 [11], [12]. Mcl-1 expression is regulated by transcription and translation [13]. Moreover, Mcl-1 has a short half-life, the result of its proteasomal degradation after ubiquitination [14], [15], which is mediated by the ubiquitin E3 ligase Mule (Mcl-1 ubiquitin ligase E3 (Mule)/ARF-BP1) [16]. Mcl-1 can also be degraded by caspases [17] and potentially by other proteases [16]. During pneumococcal infection Mcl-1 downregulation is regulated post-transcriptionally with evidence of enhanced ubiquitination [12].

Induction of macrophage apoptosis by pneumococcal infection requires internalization and killing of bacteria, an event localized to the phagolysosome [10], [18], [19]. Lysosomal membrane permeabilization (LMP) can trigger either apoptosis (through activation of lysosomal proteases of the cathepsin family) [20] or non-apoptotic cell death with features of necrosis [21], especially when LMP is extensive [22]. Cathepsins can cleave Bcl-2 family members to trigger the mitochondrial pathway of apoptosis [23], [24], [25], [26] or may directly activate caspases [27]. Despite the importance of lysosomes in antibacterial host defense, LMP has not yet been investigated in the host-pathogen relationship or linked to innate immune responses [22].

Here, we demonstrate that pneumococci trigger LMP and activation of cathepsin D in macrophages. Activation of cathepsin D enhances the interaction of Mcl-1 with its ubiquitin ligase, resulting in its destruction. The induction of macrophage apoptosis that results from cathepsin D activation provides a late increment to bacterial killing. These results indicate that cathepsin D plays a major pathophysiological role in the inter-relationship between intracellular pneumococci and macrophages that defines innate immune competence.

## Results

### Pneumococcal infection triggers lysosomal membrane permeabilization (LMP)

**Figure 1:**
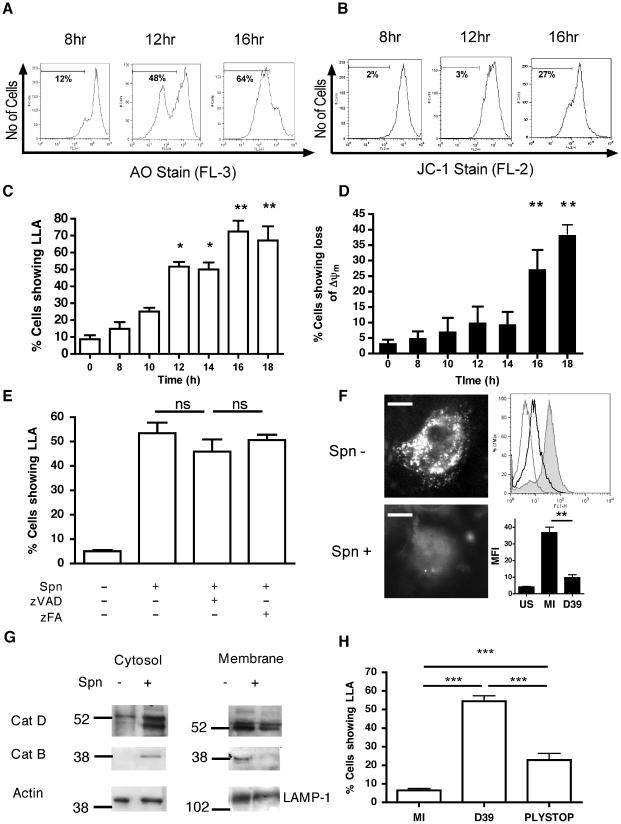
Differentiated THP-1 cells infected with pneumococci exhibit loss of lysosomal acidification and cytosolic translocation of cathepsin D. Differentiated THP-1 cells were infected with pneumococci (D39) and stained with (A, C) acridine orange (AO) and (B, D) JC-1 at the designated times post infection. (A–B) Representative histograms from one infection and (C–D) graphs summarizing loss of lysosomal acidification (LLA) and inner mitochondrial transmembrane potential (ΔΨ_m_) in three separate experiments are shown, * = p<0.05, ** = p<0.01, one way ANOVA with Dunnett's post-test *vs.* 0 h. (E) AO staining 16 h post-infection of mock infected (Spn −) or pneumococcal infected (Spn+) differentiated THP-1 cells in the presence (+) or absence (−) of zVADfmk (zVAD) or zFAfmk (zFA), n = 3. Spn+ without zVAD/zFA *vs.* Spn+ with zVAD, p = ns (not significant) (F) Mock (Spn−) or D39 (Spn+) infected cells were stained with BODIPY FL-Pepstatin A 16 h post-infection and either visualized by microscopy or analyzed by flow cytometry. The filled histogram is Spn−, black histogram is Spn+, grey is unstained (US). The images and flow histograms are representative of three independent experiments. Scale bar equal to 5 µm. Quantified flow results are shown below the histogram, n = 3 (G) A Western blot of cytosolic and membrane fractions from mock (Spn−) or D39 (Spn+) infected differentiated THP-1 cells at 16 h post-infection probed with anti–cathepsin D (CatD) and cathepsin B (CatB). Actin and LAMP-1 were used as loading controls. The blots are representative of three independent experiments. (H) AO staining of differentiated THP-1 cells 16 h after mock-infection (MI) or exposure to D39 or a pneumolysin-deficient strain of D39 (PLYSTOP), n = 4, *** p<0.001, one-way ANOVA with Tukey's post-test. In all cases, pooled data are expressed as mean +/− SEM.

Since the apoptotic program can be initiated by several organelles including lysosomes [20], we investigated whether bacterial killing in phagolysosomes was associated with signs of LMP, an early feature of some apoptotic pathways. We confirmed internalization of pneumococci in the differentiated THP-1 macrophage-like cell line (Figure S1), which we have recently shown has a similar susceptibility to apoptosis and produces similar innate responses to monocyte-derived macrophages (MDM) [28]. As early as 10 h after infection an increased percentage of cells exposed to pneumococci exhibited reduced incorporation of the acidophilic dye acridine orange, indicating loss of lysosomal acidification (LLA) (Figure 1A,C). Simultaneous staining of a separate aliquot of cells from the same cultures demonstrated that LLA occurred prior to the dissipation of the inner mitochondrial transmembrane potential (ΔΨ_m_) (Figure 1B,D). We have previously demonstrated that macrophage apoptosis during pneumococcal infection is caspase-dependent [18] and caspase activation has been reported to trigger LMP [29]. Nonetheless, addition of the broad-spectrum caspase inhibitor zVADfmk failed to prevent LLA, indicating that LLA is not a late consequence of apoptosis (Figure 1E). An alternative marker of lysosomal integrity, pepstatin A-BODIPY FL, whose binding to the lysosomal protease cathepsin D is pH-dependent [30], failed to stain the lysosomes from the infected macrophage-like cell line, while control cells exhibited a punctate lysosomal staining pattern under the same experimental conditions (Figure 1F). The lysosomal nature of staining was confirmed since organelle purification using discontinuous sucrose gradients confirmed initial cathepsin D localization in fractions stained with lysosomal markers 6 h after exposure to pneumococci (Figure S2). These results indicate that pneumococcal infections cause impairment of lysosomal acidification and/or LMP. Subcellular fractionation followed by immunoblotting revealed cytosolic translocation of cathepsins D and B in the infected macrophage-like cell line, while the amount of cathepsin D and B contained in the lysosomal fraction declined at the later time point of 16 h after exposure to pneumococci (Figure 1G). Imaging of individual cells, as shown in Figure 1F, confirmed that loss of LLA/LMP was occurring in single cells, not just at the level of the total cell population, and that LLA/LMP preceded ΔΨ_m_ and nuclear fragmentation (data not shown). The pneumococcal toxin pneumolysin was required for LLA since a pneumolysin deficient pneumococcal strain, PLYSTOP, did not induce LLA (Figure 1H), despite being internalized to a similar extent to the wild-type strain (Figure S1). Moreover complementation of this mutant with pneumolysin restored LLA, ΔΨ_m_ and cytolytic activity (Figure S3A–C). Altogether, these results indicate that pneumococci trigger LMP.

### Pneumococcal infection is associated with activation of cathepsin D

**Figure 2:**
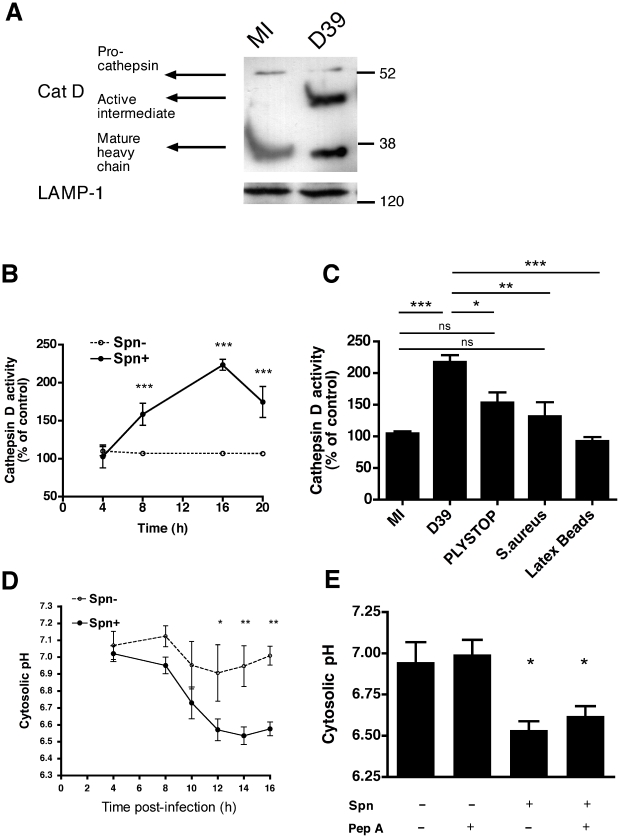
Infection with pneumococci is associated with activation of cathepsin D in differentiated THP-1 cells. (A) A Western blot of phagolysosomes prepared from differentiated THP-1 cells 14 h after mock infection (MI), or infection with *S. pneumoniae* strain D39 and isolated using a discontinuous sucrose gradient was probed for cathepsin D. The blot is representative of three independent infections. (B) Cathepsin D activity was measured in whole cell lysates from mock (Spn−) or D39 (Spn+) infected differentiated THP-1 cells at the designated time points. D39 infected cells showed elevated cathepsin D activity compared to mock infected cells from 8 h, n = 4, *** = p<0.001, two-way ANOVA (C) Cathepsin D activity measured in whole cell lysates at 14 h in mock-infected (MI) cells, or differentiated THP-1 cells infected with the designated Spn strains (D39 or the pneumolysin-deficient strain PLYSTOP), *Staph. aureus* or latex beads, n = 4, ns =  not significant * = p<0.05. ** = p<0.01, *** = p<0.001 one-way ANOVA with Tukey's post-test. (D) Cytosolic pH was measured in mock (Spn−) or D39 (Spn+) infected cells at the designated time points using SNARF-4F carboxylic acid, acetoxymethyl ester, acetate, n = 4, * = p<0.05. ** = p<0.01, two-way ANOVA. (E) Cytosolic pH was measured at 14 h in differentiated THP-1 cells either mock-infected (Spn−) or exposed to D39 pneumococci (Spn+) in the presence (+) or absence (−) of pepstatin A (Pep A), n = 4, * = p<0.05, one-way ANOVA with Dunnett's post-test *vs.* MI. In all cases, pooled data are expressed as mean +/− SEM.

Cathepsin D, a lysosomal protease, can induce apoptosis when it is activated and released into the cytosol [22]. As shown in Figure 2A, cathepsin D, the most abundant cathepsin in differentiated macrophages [31], underwent proteolytic maturation in phagolysosomes following pneumococcal infection, as evidenced by detection of the heavy chain form of active cathepsin D. We also confirmed that the organelles isolated on a sucrose gradient were phagolysosomes by identifying markers of phagolysosomes such as LAMP-1, rab-5 and -7 (Figure S2). A functional assay, based on the proteolytic processing of a fluorogenic cathepsin D substrate, confirmed that pneumococcal infection of macrophages resulted in enhanced cathepsin D activity as early as 8 h post-infection (Figure 2B), provided that the pneumococci expressed the toxin pneumolysin (Figure 2C). The pneumolysin deficient pneumococcal strain, PLYSTOP, stimulated significantly less cathepsin D activation than the isogenic wild-type strain from which it was derived. Reintroduction of pneumolysin into the PLYSTOP mutant restored activation of cathepsin D to a level comparable to the wild-type strain (Figure S3D). The cathepsin D activity was not significantly enhanced after phagocytosis of latex beads or of another Gram-positive bacterium *Staphylococcus aureus*, which is readily internalized [32]. Cathepsin D activity is optimal at acidic pH, and bacterial phagocytosis can result in cytosolic acidification [33], [34]. We found the cytosolic pH was acidified following pneumococcal infection (Figure 2D–E). The reduction in cytosolic pH occurred with the same kinetics as LLA (Figure 1C), and before dissipation of ΔΨ_m_ (Figure 1D). A cathepsin D inhibitor, pepstatin A, blocked cathepsin D activation (Figure S3D) but failed to reverse the reduction in cytosolic pH of cells exposed to pneumococci (Figure 2E), indicating that the cytosolic acidification was not a consequence of cathepsin D activation. Altogether our data suggest LMP allows release of active cathepsin D into an acidified cytosol.

### Cathepsin D activation is required for macrophage apoptosis during pneumococcal infection

**Figure 3:**
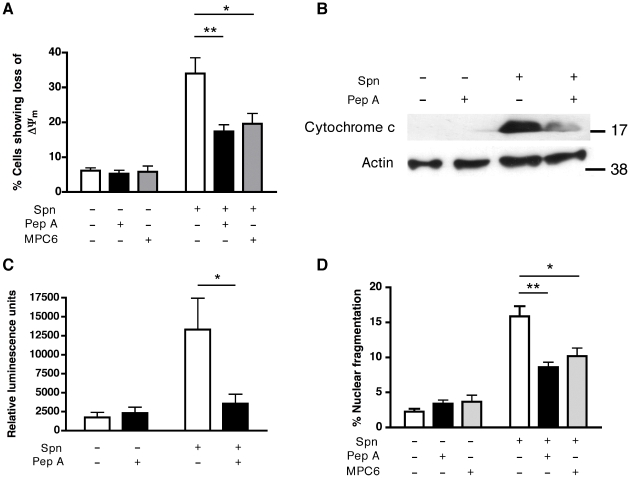
Cathepsin D activity contributes to apoptosis in the differentiated THP-1 cell line. (A) Differentiated THP-1 cells were stained with JC-1 16 h after mock-infection (Spn−) or exposure to D39 pneumococci (Spn+) in the presence (+) or absence (−) of pepstatin A or MPC6. Loss of fluorescence indicates loss of ΔΨ_m_, n = 3–5, * = p<0.05, ** = p<0.01, two-way ANOVA. (B) A representative Western blot of the cytosolic fractions of Spn− or Spn+ cells, 16 h after infection, in cultures incubated with (+) or without (−) pepstatin A (PepA). The blot is representative of four independent infections. (C) Spn- or Spn+ differentiated THP-1 cells, incubated in the presence (+) or absence (−) of pepstatin A (PepA), were assayed for caspase activity by fluorimetry 16 h post-infection, n = 5, * = p<0.05. (D) Spn− or Spn+ differentiated THP-1 cells, incubated in the presence (+) or absence (−) of pepstatin A or MPC6, were fixed and analyzed for nuclear fragmentation after 20 h culture, n = 3–4, * = p<0.05, ** = p<0.01, two-way ANOVA. In all cases, pooled data are expressed as mean +/− SEM.

A range of inhibitors active against cathepsins B, D and L, the most abundant cathepsins in differentiated macrophages [35], were screened for their capacity to prevent loss of ΔΨ_m_, one of the first signs of irreversible cell death. Only inhibitors with activity against the aspartic protease cathepsin D (but not B or L) were able to prevent the dissipation of ΔΨ_m_ (Figure S4A). Pepstatin A inhibited loss of ΔΨ_m_ (Figure 3A) and prevented the mitochondrial cytochrome *c* release induced by pneumococcal infection (Figure 3B). Pepstatin A also inhibited other signs of apoptosis including caspase 3/7 activation, chromatin condensation and nuclear fragmentation (Figure 3C–D). The anti-apoptotic activity of pepstatin was shared with other cathepsin D inhibitors, such as MPC6 (Figure 3A and 3D) and DAME (Figure S4B). Pepstatin A inhibited apoptosis in the macrophage-like cell line, and the residual apoptosis was further blocked by an anti-oxidant and an inhibitor of inducible nitric oxide synthase (Figure S5). The key findings of cathepsin D activation, LLA and reduced apoptosis (dissipation of ΔΨ_m_ and nuclear fragmentation) with pepstatin A treatment, following pneumococcal infection, were replicated in monocyte-derived macrophages (MDM; Figure S6). These results suggest cathepsin D plays a critical role in macrophage apoptosis during pneumococcal infection, downstream of LMP but upstream of the mitochondrial phase of the cell death pathway.

### Cathepsin D-deficient macrophages are resistant to apoptosis during pneumococcal infection

**Figure 4:**
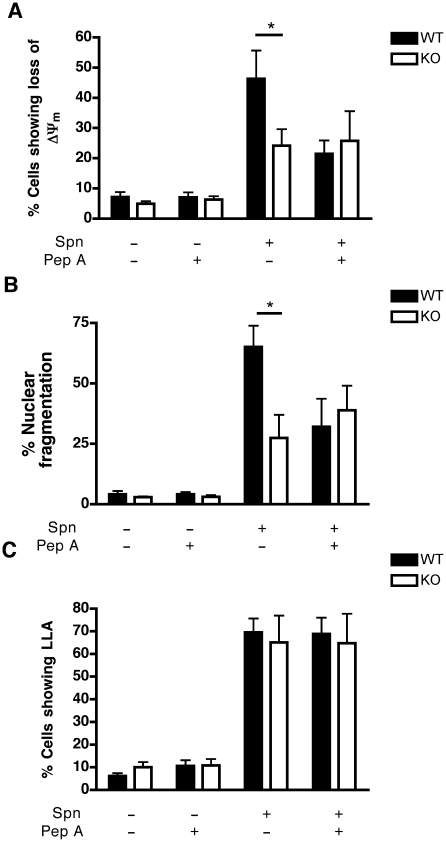
Macrophages deficient in cathepsin D show reduced apoptosis. (A) BMDM's from (WT) or cathepsin D knock-out (KO) mice were stained with JC-1, 16 h after mock-infection (Spn−) or pneumococcal infection (Spn+) with D39 in the presence (+) or absence (−) of pepstatin A (PepA), n = 7, * = p<0.05, two-way ANOVA. (B) Nuclear fragmentation was detected by DAPI staining, in WT and cathepsin D KO BMDMs, 20 h after mock (Spn−) or D39 pneumococcal (Spn+) infection in the presence (+) or absence (−) of PepA, n = 5 per group. (C) Acridine orange staining of BMDMs 16 h post-infection in the presence (+) or absence (−) of pepstatin A, n = 7 per group, * = p<0.05, two-way ANOVA. In all cases, pooled data are expressed as mean +/− SEM.

To exclude off-target effects of pharmacological inhibitors and since cathepsin D is the major aspartic protease inhibited by pepstatin A, but other aspartic proteases could also be inhibited [36], bone marrow-derived macrophages (BMDM) were generated from mice from which the gene encoding cathepsin D was deleted or from wild-type (WT) littermates. WT and cathepsin D^−/−^ BMDM exhibit comparable lysosomal density and internalize similar numbers of opsonized pneumococci (Figure S7A–B). Following pneumococcal infection, cathepsin D^−/−^ BMDM failed to demonstrate similar levels of apoptosis under conditions that caused WT BMDM to undergo dissipation of ΔΨ_m_ (Figure 4A), chromatin condensation and nuclear fragmentation (Figure 4B). Addition of pepstatin A to WT BMDM infected with pneumococci phenocopied the cathepsin D^−/−^ genotype as far as the protection of mitochondrial and nuclear integrity were concerned (Figure 4A–B). However, there was no difference between untreated WT BMDM, pepstatin-treated BMDM or cathepsin D^−/−^ BMDM at the level of LLA induced by pneumococcal infection (Figure 4C). These results support the conclusion that cathepsin D operates downstream of LLA but upstream of the mitochondrial cell death pathway.

### Cathepsin D enhances Mcl-1 ubiquitination

**Figure 5:**
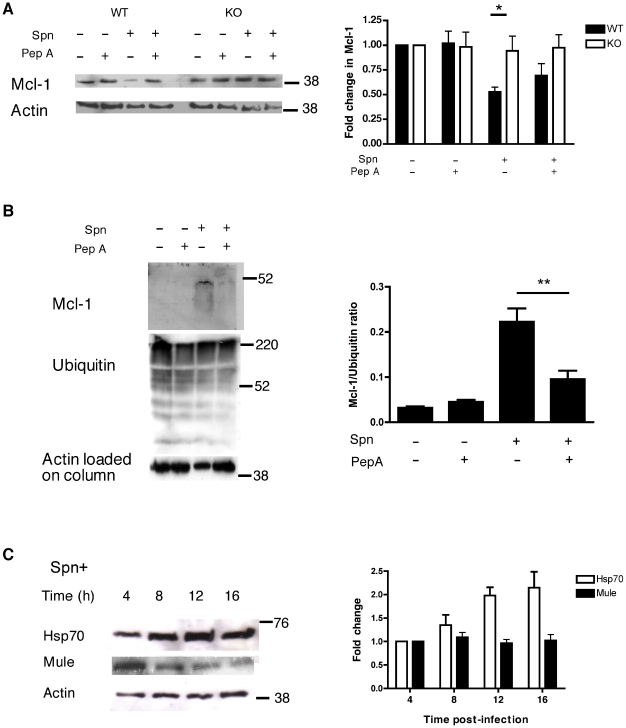
Cathepsin D facilitates Mcl-1 downregulation. (A) A representative Western blot for Mcl-1 from wild-type (WT) and Cathepsin D knock-out (KO) BMDMs 16 h after mock- (Spn−) or D39 pneumococcal-infection (Spn+), in the presence (+) or absence (−) of pepstatin A. The blot is representative of four independent experiments. Densitometry was carried out and fold change of Mcl-1 relative to mock-infection was calculated, n = 4 * = p<0.05, two-way ANOVA. (B) Spn− or Spn+ differentiated THP-1 cells were cultured in the presence (+) or absence (−) of pepstatin A (PepA). At 16 h cells were lysed, ubiquitinated proteins captured, and Western blots carried out probing for total ubiquitinated proteins and for Mcl-1. Densitometry was carried out and the ratios of Mcl-1 relative to ubiquitin were calculated, n = 3, * = p<0.05, one-way ANOVA with Tukey's post-test. (C) Spn− or Spn+ differentiated THP-1 cells were lysed and probed for Hsp70, Mule and actin at the designated times post-infection. The blots are representative of three experiments and the results summarized by densitometry.

WT BMDM showed a reduction in Mcl-1 protein levels after pneumococcal infection, as previously described [12]. This effect was reversed by treatment with pepstatin A or in cathepsin D^−/−^ BMDM (Figure 5A). Pepstatin A treatment also reduced the loss of Mcl-1 following pneumococcal infection in MDMs (data not shown). Although some proteins involved in apoptosis induction, such as caspase 8, are direct cathepsin D substrates [27], we found no evidence Mcl-1 was a cathepsin D substrate, either by *in silico* analysis [37] or by searching for Mcl-1 cleavage products in overexposed immunoblots (data not shown). In contrast, we observed that pneumococcal infection enhanced the ubiquitination of Mcl-1 and that cathepsin D inhibition reversed this process (Figure 5B). Mcl-1 ubiquitination is catalyzed by Mule/ARF-BP1, an E3 ubiquitin ligase [16]. Heat shock protein Hsp70 reduces Mule binding to Mcl-1 and Mcl-1 polyubiquitination [38]. Hsp70 expression was induced but there was no evidence of induction of Mule expression following pneumococcal infection (Figure 5C).

**Figure 6:**
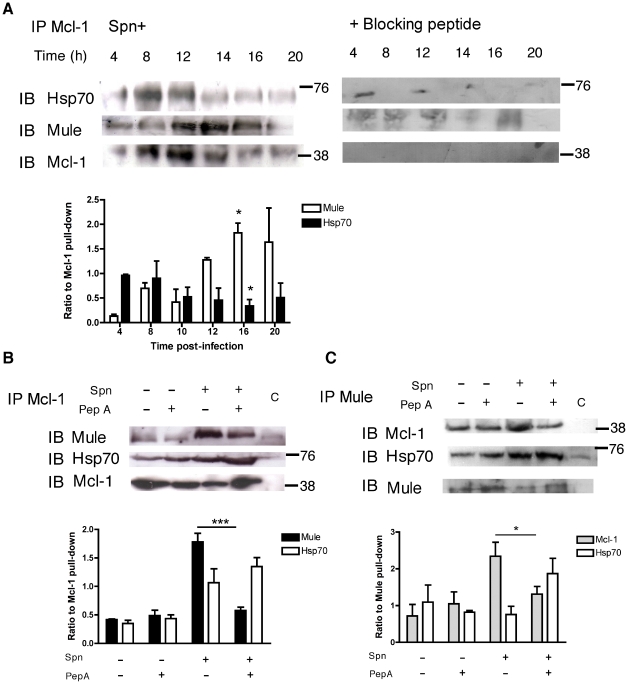
Cathepsin D activation favors the interaction between Mcl-1 and Mule. (A) Mock-infected (Spn−) or D39 exposed (Spn+) differentiated THP-1 cells were immunoprecipitated with an anti-Mcl-1 antibody at the designated time period. As controls a separate sample at each time point was treated with a Mcl-1 peptide. The peptide used was identical to that used to produce the anti-Mcl-1 antibody. This excluded non-specific binding by the anti-Mcl-1 antibody. Precipitated proteins were blotted for Hsp70, Mule and Mcl-1. Densitometry was carried out and the ratios of Mule and Hsp70 to the amount of Mcl-1 precipitated was calculated, n = 3 * = p<0.05 for comparison of 4 h *vs.* 16 h, 1-way ANOVA with Dunnett's post test. Spn− or Spn+ differentiated THP-1 cells were cultured in the presence (+) or absence (−) of pepstatin A (PepA) for 16 h and lysates were immunoprecipitated with anti-Mcl-1 (B) or anti-Mule (C) antibody before being probed for Mule, Hsp70 and Mcl-1. C represents a control in which mock-infected (MI) cells were immunoprecipitated in the presence of an excess of antigen-specific peptide (Mcl-1) or a non-specific antibody for the immunoprecipitation of Mule. Densitometry was carried out and the ratios of the immunoblotted proteins to the immunoprecipitated Mcl-1 or Mule were calculated, n = 3 *** = p<0.001 for comparison of Spn+ with or without pepstatin A, one-way ANOVA with Tukey's post-test.

Immunoprecipitation of Mcl-1 demonstrated the expected downregulation of Mcl-1 with time but indicated there was a sequential increase in Hsp70 binding (until 12 h) and in Mule binding (from 12 h) (Figure 6A). The enhancement of the Mcl-1-Mule interaction, which was triggered by pneumococcal infection, was demonstrated by immunoprecipitation of either Mcl-1 or Mule and was reversed by pepstatin A (Figure 6B–C). Conversely, following pepstatin A treatment the interaction between Hsp70 and Mcl-1 was favored (Figure 6B–C).

### Inhibition of cathepsin D decreases bacterial killing

**Figure 7:**
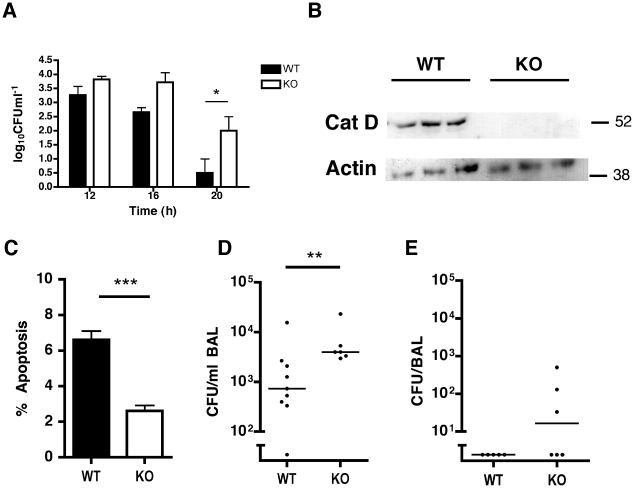
Cathepsin D in alveolar macrophages contributes to bacterial killing *in vitro* and *in vivo*. (A) BMDMs from (WT) or cathepsin D knock-out (KO) mice were exposed to D39 pneumococci for the indicated time periods. Cells were lysed and intracellular bacteria plated out at the designated times, n = 5 per group, * = p<0.05, two-way ANOVA. (B) A representative Western blot of alveolar macrophages obtained from bronchial alveolar (BAL) fluid from irradiated mice transplanted with bone marrow from cathepsin D WT or KO mice. (C) The percentage of apoptotic alveolar macrophages in BAL in mice after adoptive transfer of marrow from WT or KO mice, 24 h after infection with 10^4^ type 1 pneumococci, as assessed by nuclear morphology, n = 6–10. The number of surviving bacteria in BAL 14 h (D) and 24 h (E) after infection with 10^4^ type 1 pneumococci, n = 5–9 ** = p<0.01, *** = p<0.001, students t-test. In all cases, pooled data are expressed as mean +/− SEM.

The inhibition of macrophage apoptosis that results from maintenance of high Mcl-1 levels prevents effective bacterial killing [12]. We confirmed that caspase inhibition, which reduces macrophage apoptosis, but does not alter cathepsin D activation after pneumococcal infection, reduced bacterial killing in differentiated THP-1 cells (Figure S8). Pepstatin A also reduced bacterial killing. The combination of pepstatin A and caspase inhibitors did not further suppress the level of apoptosis nor did it further reduce bacterial killing, suggesting that the antimicrobial effect of pepstatin A was mediated via inhibition of apoptosis. Intracellular killing assays were also performed with BMDM from WT or cathepsin D^−/−^ mice. While there was no difference in bacterial colony counts early post-infection (0–10 h), we detected a 1–1.5 log increase in intracellular bacterial colony counts 16–20 h after infection in the cathepsin D^−/−^ BMDM (Figure 7A). These time points correspond to the time of induction of mitochondrial (Figure 1D) and other downstream features of apoptosis in this model [12], thus confirming a critical role for cathepsin D in the late increment to bacterial killing provided by macrophage apoptosis.

A further series of experiments were performed in irradiated mice that were reconstituted with either cathepsin D^+/+^ or cathepsin D^−/−^ bone marrow cells. These mice have normal numbers of myeloid cells including macrophages and neutrophils [39] and we also confirmed no baseline differences in numbers of apoptotic cells in the lung (data not shown). Cathepsin D^−/−^ alveolar macrophages from reconstituted mice were normal in number (Figure S9), yet exhibited absent cathepsin D expression and reduced apoptosis following pulmonary infection with pneumococci, as compared to cathepsin D^+/+^ controls (Figure 7B–C). Mice that had undergone bone marrow transplantation with cathepsin D^−/−^ bone marrow were significantly impaired in their capacity to clear low inocula of bacteria from the lungs (Figure 7D–E), in a model of subclinical infection in which alveolar macrophages ensure bacterial clearance and mouse survival [8].

### Lack of cathepsin D activation results in increased recruitment of neutrophils

**Figure 8:**
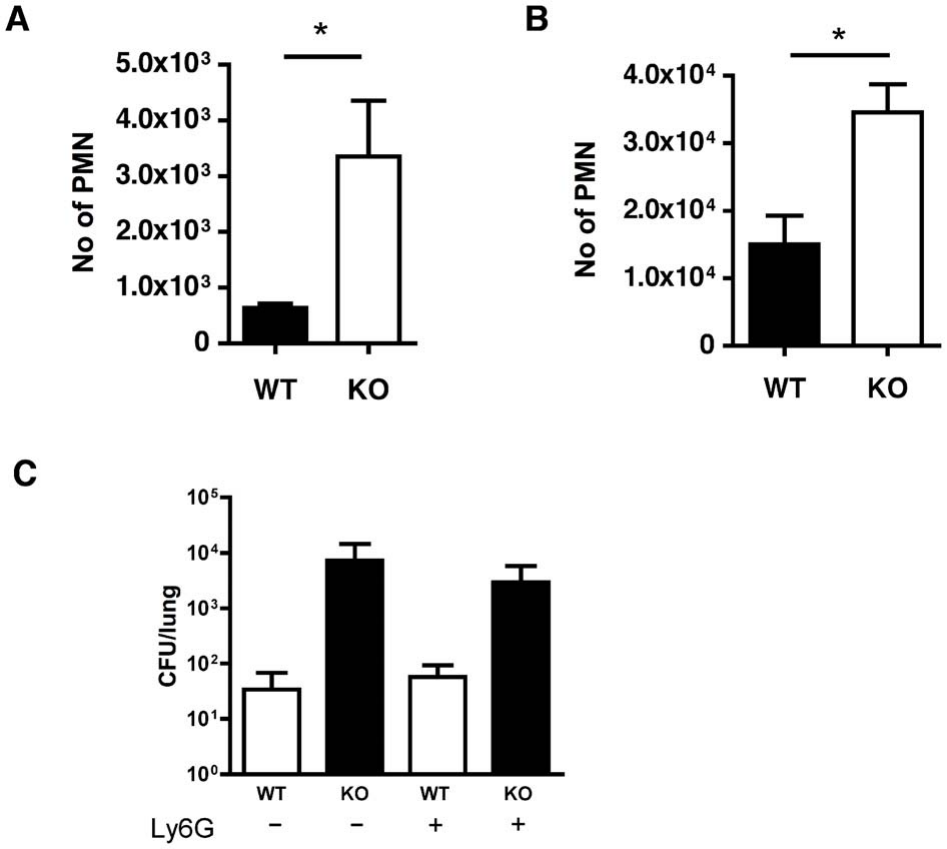
Absence of functional cathepsin D in macrophages results in increased neutrophil recruitment. Mice were transplanted with bone marrow from cathepsin D deficient (KO) mice or with bone marrow from wild-type littermates (WT). Mice were instilled with (A) 10^3^ colony forming units of type 1 pneumococci for 24 h or (B) with 10^4^ type 1 pneumococci for 14 h and the number of neutrophils in BAL was calculated by analysis of cytospins. In all experiments, n = 3–9 * = p<0.05, students t-test. (C) Mice received bone marrow transplantation as above and WT or KO mice were instilled with 10^4^ type 1 pneumococci in the presence (+) of anti-Ly6G antibody or control antibody (−) to deplete neutrophils, n = 6 per group. In all cases, pooled data are expressed as mean +/− SEM.

Both cathepsin D^+/+^ and cathepsin D^−/−^ reconstituted mice recruited neutrophils following low dose pneumococcal challenge (Figure 8A–B). This contrasts with mice which have not undergone bone marrow transplantation, which can control these levels of bacteria without neutrophil recruitment and in which impairment of macrophage mediated bacterial clearance results in enhanced neutrophil recruitment [8]. It is also consistent with the known effect of bone marrow transplantation to reduce the effectiveness of pulmonary anti-bacterial host defense [40]. However in two challenge models mice reconstituted with cathepsin D^−/−^ macrophages demonstrated significantly greater recruitment of neutrophils, a marker of reduced capacity to control infection and of more extensive disease in these low dose pneumococcal challenge models [41] than did mice reconstituted with cathepsin D^+/+^ macrophages (Figure 8A–B).

The engagement of CXCR2 by CXC chemokines is critical for neutrophil recruitment in murine models of pneumococcal pneumonia [42], [43], [44]. Preliminary data showed cathepsin D^−/−^ BMDM produced higher levels of KC and MIP-2 than WT BMDM, 16 h after bacterial exposure, consistent with a role for these chemokines in neutrophil recruitment in other models of pneumococcal pneumonia (data not shown). To establish whether the recruited neutrophils were contributing to bacterial clearance we depleted these with an anti-Ly6G antibody [45]. Mice treated with this antibody had reduced numbers of neutrophils and WT mice approximately doubled the bacterial CFU in the lungs after treatment with anti-Ly6G (Figure 8C), suggesting that at this low level of infection neutrophils were helping to clear bacteria. Nevertheless although mice reconstituted with cathepsin D^−/−^ bone marrow had significantly higher colony counts in the lung than those reconstituted with WT marrow, there was no significant difference in the bacterial colony counts in the lungs of mice reconstituted with cathepsin D^−/−^ after neutrophil depletion. This suggested that the role of neutrophils in host defense was redundant and could be compensated for by other factors, particularly for mice reconstituted with cathepsin D^−/−^ bone marrow, during low dose pneumococcal challenge.

In summary, cathepsin D was essential for apoptosis-associated pneumococcal killing and in the absence of cathepsin D expression by macrophages there was evidence of impaired bacterial clearance and markers of more extensive pulmonary disease.

## Discussion

During bacterial infection prolonged intracellular killing leads to macrophage apoptosis, a process which contributes to the late phase killing of pneumococci [8]. The onset of apoptosis is determined by the level of the anti-apoptotic protein Mcl-1, a protein whose short intracellular half-life and regulation by ubiquitination make it well suited to transducing critical levels of cell stress into a program of apoptosis [12]. In this study, we demonstrate that LMP and cathepsin D activation trigger macrophage apoptosis via Mcl-1 downregulation during pneumococcal infection. Cathepsin D activation stimulates Mcl-1 ubiquitination, correlating with enhanced binding of Mcl-1 to its ubiquitin ligase, Mule. Cathepsin D is required for host-mediated macrophage apoptosis and the apoptosis-associated late phase of bacterial killing. Moreover mice reconstituted with cathepsin D^−/−^ bone marrow have significant impairment in their capacity to clear pneumococci from the lung and recruit greater numbers of neutrophils, the central pathologic feature of pneumococcal pneumonia.

To our knowledge, activation of lysosomal death pathways during phagolysosomal killing of microorganisms, has not been previously linked to programs of apoptosis, despite the fact that LMP and cathepsins have been implicated in multiple cell death scenarios [20], [21], [23], [25], [46], [47]. The delayed macrophage death seen during pneumococcal infection is associated with ΔΨ_m_ dissipation, mitochondrial cytochrome *c* release, caspase activation and nuclear fragmentation, which are all hallmarks of classical apoptosis [12], [18], [48]. We previously observed that macrophage apoptosis during pneumococcal killing requires the cholesterol-dependent cytolysin pneumolysin [48], [49]. Since cholesterol-dependent cytolysins can bind host proteins including pattern recognition receptors [50], [51], and a diverse range of bacteria can trigger an apoptotic response during bacterial clearance [6], it is likely that recognition of pneumolysin is part of a more general innate response which links detection of different bacterial proteins to LMP, cathepsin D activation and subsequent apoptosis induction. Cathepsin D may contribute to the recognition of these bacterial factors by playing a role in their processing. The cholesterol-dependent cytolysin, listerolysin, is cleaved by cathepsin D [52]. However using HIS-tagged pneumolysin, we have so far been unable to confirm any direct interaction between pneumolysin and cathepsin D (Bewley and Dockrell unpublished observations).

Although the molecular mechanisms of LMP in apoptosis are incompletely characterized, potential causes in our model include generation of lysosomotropic factors such as ceramide, intracellular calcium flux or reactive oxygen species [53]. Moreover we clearly demonstrate LLA and cathepsin D activation occur upstream of the mitochondrial features of cell death and are not the result of caspase activation [29]. We have previously shown that the mitochondrial apoptosis pathway is only activated when Mcl-1 expression in macrophages falls below maximal levels during pneumococcal infection [12], [48].

We evaluated several cathepsins but observed that cathepsin D was the major mediator of macrophage apoptosis induced during pneumococcal infection, though not the only factor as evidenced by the fact that absence of cathepsin D activation did not completely abolish apoptosis. Other minor factors are likely to include other proteases and protease stress is likely to interact with oxidative and, as we have previously shown, nitrosative stress [48]. In keeping with this, the combination of an antioxidant, inhibition of inducible nitric oxide synthase and pepstatin A treatment was particularly efficient in suppressing apoptosis. However, as the bulk of the inhibition was achieved with each agent alone, we surmise that all these inhibitors act on a common pathway that converges at the level of the mitochondria. Cathepsin D is the most abundant cathepsin in differentiated macrophages[31], [54]. The delayed process of cell death we have observed following pneumococcal infection is differentiation-dependent [10], commensurate with the accumulation of lysosomes and of cathepsin D in differentiated macrophages [31], [55]. We found that pneumococcal infection activated cathepsin D, while phagocytosis of *Staph. aureus* failed to do so. The observation that *Staph. aureus*, which is readily phagocytosed [32], fails to result in significant activation of cathepsin D was noteworthy since *Staph. aureus* is known to survive in macrophages and prevent macrophage apoptosis by upregulating Mcl-1 [56].

Cathepsin D was found to be activated within phagolysosomes and may exert its pro-apoptotic effects either on substrates in the phagolysosome or in the cytosol after translocation from phagolysosomes. A fall in cytosolic pH has been identified as a consequence of bacterial phagocytosis and killing in phagocytes [33], [34]. LLA allows dissociation of procathepsin D from the phagolysosomal membrane, cathepsin D activation and substrate cleavage in the phagolysosomal lumen [57]. It has been assumed that cathepsin D has little activity above pH 6.2 and that its role in the cytosol would be non-catalytic [58]. However, the pH dependence of cathepsin D activity is substrate-dependent and residual activity is detected for some substrates at pH above 6 [59]. Alternatively, as proposed by Conus, acidic pH dependent-activity may be retained in the vicinity of permeabilized phagolysosomes [27]. Although, we cannot exclude the possibility that some intermediary molecule was cleaved by cathepsin D, we found no evidence that Mcl-1 itself was a substrate. Cathepsin D exerts some of its functions such as mitogenic stimulation or modulation of apoptosis by non-catalytic activity [22], [58]. Nonetheless, the ability of cathepsin D inhibitors to prevent the downstream effects, as well as the persistent cathepsin D activity in macrophages exposed to pneumococci, argues for an important role for cathepsin D acting as a protease in this model.

Cathepsin D had indirect effects on Mcl-1 regulation via enhanced ubiquitination of Mcl-1, a major mechanism of Mcl-1 degradation [15], consequent upon increased association of Mcl-1 with its ubiquitin ligase, Mule [16]. The mechanism through which the Mcl-1/Mule interaction is increased requires further elucidation, but could include the cathepsin D-dependent activation of BH3-only proteins, releasing Mcl-1 to interact with Mule. We found no evidence that cathepsin D activation reduced levels of Hsp70 protein, a further factor competing for Mcl-1 binding to Mule [38]. Hsp70 is likely to contribute to the maintenance of macrophage cell viability during the first 12 h after exposure to pneumococci as it prevents both LMP and Mcl-1 ubiquitination [38], [60].

Our demonstration of activation of cathepsin D prior to induction of a mitochondrial pathway of apoptosis has provided us with a new tool with which to probe the function of macrophage apoptosis in host defense against pneumococci. Cathepsin D did not contribute to bacterial killing prior to apoptosis induction and pepstatin A did not reduce bacterial killing beyond the level observed with a caspase inhibitor. Moreover, by using a murine model in which alveolar macrophages were the only resident cells that had resistance to apoptosis, we are able to clarify the role of macrophage apoptosis in a fashion not previously possible with approaches using caspase inhibition or Mcl-1 over-expression, in which apoptosis resistance is not selective [8], [12]. Using this approach we now clearly show that macrophage apoptosis is required for late phase bacterial killing. Resident components of host defense in the lung control small numbers of bacteria that penetrate the distal airway. When macrophage function is subverted by the pathogen [61] or by the sheer size of the bacterial inoculum [10], activation of apoptosis facilitates bacterial killing. We cannot at present state the exact basis of this observation. It could reflect release of factors during the apoptotic process, such as mitochondrial ROS, that have antimicrobial effects, a dual role for an effector of apoptosis induction in also contributing to antimicrobial killing or the possibility that apoptotic bodies contain bacteria that are then killed by other macrophages when these bodies are efferocytosed. This study confirms that cathepsin D contributes to the antimicrobial effect of macrophage apoptosis during host defense against pneumococci. We speculate that the induction of apoptosis allows containment of bacteria when the phagolysosomal antimicrobial capacity is ‘exhausted’ and prevents bacterial persistence within subcellular compartments that lack antimicrobial capacity. When the bactericidal contribution of macrophage apoptosis is overwhelmed, additional effectors including neutrophils are activated, and when the inoculum is low, most bacteria are cleared through redundant mechanisms. Increasing the inoculum overwhelms these overlapping but redundant elements of host defense and allows transition to established pneumonia [8]. Macrophage apoptosis therefore benefits the host at the critical transition between sub-clinical infection and establishment of pneumonia, yet has a finite capacity to control infection and can be overwhelmed by a large bacterial challenge.

In conclusion, we provide evidence that pneumococci stimulate macrophage LMP. We have found that cathepsin D is a central effector of apoptosis and that its activation and lysosomal release functions as a ‘danger signal’ which alerts macrophages to LMP and the potential translocation of bacteria into the cytosol. Enhanced ubiquitination of Mcl-1 results in its depletion, thus initiating the mitochondrial pathway of apoptosis (Figure S10). Cathepsin D is not only the trigger for apoptotic death of infected macrophages but is required for the optimal clearance of pneumococci, at the critical transition between sub-clinical infection and establishment of pneumonia, supporting an intimate functional relationship between apoptosis of macrophages and their bactericidal activity.

## Materials and Methods

### Bacteria

Type 2 (D39 strain, NCTC 7466) and mutant strain (PLYSTOP) of *S. pneumoniae* (Spn) or type 1 (SSISP1/1) for murine experiments (at the indicated inocula) were grown as described [8]. *Staphylococcus aureus* (strain SH1000) was grown up in Brain Heart Infusion (BHI) supplemented with 20% v/v FCS until an OD_610 nm_ of 0.6 was reached. Prior to infection with *S. pneumoniae* strains, thawed aliquots were opsonized in RPMI (Sigma-Aldrich) containing 10% v/v anti-pneumococcal immune serum [18]. Bacterial numbers were assessed by the surface viable count method after inoculation on blood agar.

### Construction of PLYSTOP

A version of D39, in which toxin production was interrupted by introduction of a translational stop codon at the 5′ end of the gene, was made by first inserting an extra T base after base 6 in the pneumolysin gene by site-directed mutagenesis. The altered gene was then introduced into the chromosome using Janus mutagenesis [62]. The resultant strain was shown not to produce any toxin as judged by Western blotting and lack of haemolytic activity and findings were confirmed using a complementation mutant (Figure S3). The complementation mutation was constructed by transformation with the shuttle vector pALYI [63] containing the appropriate DNA insert.

### Cells and infection

THP-1 cells were cultured in RPMI plus 10% v/v FCS (complete media). THP-1 cells were differentiated to a macrophage phenotype by treating 0.4×10^6^ cell/ml with 200 nM PMA for 3 d, after which the PMA was removed, and the cells left to rest for a further 5 d after which cell concentration was determined. These cells have a phenotype similar to monocyte-derived macrophages (MDM), as evidenced by nuclear to cytoplasmic ratio, concentration of mitochondria and lysosomes, cell surface markers, phagocytic capacity, cytokine generation to Toll-like receptor agonists and susceptibility to apoptosis [28]. Key findings were also repeated in MDM prepared as described [18]. Murine BMDMs were obtained by culturing marrow from mice deficient in either cathepsin D [39], or from the corresponding wild-type littermates. BMDMs were plated at 0.5×10^6^ cells/ml for 14 d in DMEM containing 10% FCS and 10% conditioned L929 media [8]. After 14 d, representative wells were scraped to determine cell concentration. All cell types were infected with opsonized pneumococci or *Staph. aureus* at a multiplicity of infection of 10, or mock-infected as described [18]. All pneumococcal strains where shown to be internalized at similar rates (Figure S1). In some experiments, cells were incubated with either 100 µM of the aspartic protease inhibitor pepstatin A, 10 µM of mannose-pepstatin conjugate (MPC) 6 [64], 2 µM of the cathepsin D inhibitor diazoacetyl-DL-2-aminohexanoic acid-methyl ester (DAME) (Bachem), 25 µM of the cathepsin B inhibitor CA-074Me (Sigma), 50 µM of the cathepsin B and L inhibitor E-64d (Sigma), 50 µM N-Benzyloxycarbonyl-Val-Ala-Asp(O-Me) fluoromethyl ketone zVADfmk (Enzymes Systems Products) as a pan-caspase inhibitor previously demonstrated to inhibit caspase-dependent macrophage apoptosis or 50 µM *N*-benzyloxycarbonyl–Phe-Ala fluoromethyl ketone, (zFAfmk) (Enzyme Systems Products) as a control for zVADfmk [18], 50 µM trolox (Calbiochem) as an anti-oxidant and 50 µM 1400 W (Calbiochem) as a specific iNOS inhibitor [48]. Cells were treated for 1 h before infection, and again from 4 h (after washing). Chemokines were measured in BMDM supernatants using a cytokine ELISA as previously described [65]

### Isolation and identification phagolysosomes

Phagolysosomes were isolated using discontinuous sucrose gradients [66]. At the designated time-point infected cells were washed three times in PBS, before being scraped and pelleted. Cells were then washed and re-suspended in homogenization buffer (250 mM sucrose, 0.5 mM EGTA, 20 mM Hepes), and homogenized on ice in a Dounce homogenizer, confirming lysis by light microscopy. The resulting lysate was cleared of unlysed cells and nuclei by centrifugation at 4°C at 450 *g* for 5 min. The phagolysosomes were then isolated by flotation on a sucrose gradient (all sucrose solutions w/v in 0.5 mM EGTA, 20 mM Hepes); the phagolysosome containing supernatant was first adjusted to 39% sucrose by addition of 65% sucrose solution. This 39% sucrose supernatant was pipetted into an ultracentrifuge tube containing 1 ml 65% sucrose overlaid with 2 ml 55% sucrose. On top of the sample 2 ml steps of 32.5% and 10% sucrose were added. The resulting five step gradient was spun for 1 h at 4°C at 100,000 *g* (SW40Ti rotor in a Beckman centrifuge). Latex bead (Sigma-Aldrich) containing phagolysosomes were collected from the interface of the 10% and 32.5% solutions. Bacteria containing phagolysosomes were isolated from the 55%/65% interface. Latex bead containing phagolysosomes were then added to PBS and pelleted by spinning for 15 min at 40,000 *g*. Bacteria containing phagolysosomes were equilibrated to 11% sucrose (using homogenization buffer without sucrose), and overlaid on a 15% Ficoll cushion (in 5% sucrose, 0.5 mM EGTA, 20 mM Hepes) and spun for 20 min at 18,000 *g*. The resulting pellet was then re-suspended in homogenization buffer and spun for 10 min at 18,000 *g*. For positive identification of phagolysosomes on the initial gradient, after the first centrifugation step the sucrose gradient was aliquoted into fractions and the protein in each aliquot TCA precipitated. Each fraction was probed for proteins known to be associated with phagolysosomes and visualised by Western blot. Each fraction was probed with anti-pneumolysin (1∶1000, provided by T. Mitchell), cathepsin D (1∶1000, R&D systems), rab5 (mouse monoclonal, 1∶1000, BD Bioscience), rab7 (mouse monoclonal, 1∶1000, Abcam), or LAMP-1 (mouse monoclonal, 1∶1000, BD Bioscience). Each fraction was also probed for the Golgi protein GM130 (mouse monoclonal 1∶1000, BD bioscience).

### SDS-PAGE and Western immunoblotting

Whole cell extracts and cytosolic fractions were isolated as previously described [48]. Blots were incubated overnight at 4°C with antibodies against either human Mcl-1 (rabbit polyclonal SC-19, 1∶1000; Santa Cruz Biotechnology Inc, recognizing full length Mcl-1, 40 kDa and ubiquitinated Mcl-1, >40 kDa), murine Mcl-1 (1∶1000; Rockland), cytochrome *c* (mouse monoclonal, 1∶1000; BD Biosciences), cathepsin D (goat polyclonal, 1∶1000; R&D Systems, recognizing pro-cathepsin D (52 kDa), an active intermediate (48 kDa) and the heavy chain of active cathepsin D (34 kDa)), cathepsin B (mouse monoclonal, 1∶1000, Abcam), actin (rabbit polyclonal 1∶5000; Sigma-Aldrich), Mule/ARF-BP1 (1∶500, Abcam), Hsp70 (rabbit polyclonal, 1∶1000; Abcam), LAMP-1 (mouse monoclonal, 1∶1000; BD Bioscience) or ubiquitin (Pierce Scientific 1∶500). Protein detection was with horseradish peroxidase conjugated secondary antibodies (1∶2000; Dako) and ECL (Amersham Pharmacia). Bands were quantified using Image J 1.32 software (NIH). In Western blot experiments fold change from mock-infected or earliest time-point was calculated and normalized to the fold change in actin [12]. In co-immunoprecipitation experiments the ratio of the blotted proteins to the precipitated protein was calculated.

### Immunoprecipitations

For IPs, cells were lysed in 2% 3-[(3-cholamidopropyl) dimethylammonio]-1-propane sulfonate hydrate (CHAPS) lysis buffer (20 mM Tris-HCl (pH 7.4), 137 mM NaCl, 2 mM EDTA, 2% CHAPS) containing phosphatase and protease inhibitors (2 mg/ml each of pepstatin, leupeptin and aprotinin) and phosphatase inhibitors (50 mM sodium fluoride and 1 mM sodium vanadate) for 30 min on ice. The lysates were incubated overnight at 4°C on a rotator with 2 µg of anti-Mcl-1 (sc-819: Santa Cruz) antibody or anti-Mule (Abcam). Immunoprecipitates were collected by the addition of 10 µl of washed protein A agarose beads (EZview Affinity Gel; Sigma-Aldrich) and incubation for 1 h at 4°C on a rotator. The beads were collected by centrifugation and washed three times with lysis buffer. Finally, the pelleted beads were resuspended in sodium dodecyl sulfate (SDS) sample buffer and heated at 95°C for analysis by SDS-polyacrylamide gel electrophoresis, loading equal concentrations of protein from the original lysate, and Western blotting with the stated antibodies. The specificity of the co-IP results was tested by performing Mcl-1 IPs in the presence of an excess of the Mcl-1 peptide, which had been used to generate the antibody (sc-819P: Santa Cruz Biotechnology) and the mule IPs with a non-specific antibody (rabbit IgG, Sigma).

### Lysosomal immunocytochemistry

BMDM were fixed for 15 min in 2% paraformaldehyde, permeabilized with 0.2% Triton-X for 5 min, before being stained using standard protocols for indirect immunofluorescence. LAMP-1 was visualized with anti-LAMP-1 antibody (1∶100, BD Bioscience) and rabbit anti-mouse FITC (DAKO). Slides were counterstained with DAPI (blue) to show nuclear localization.

### Analysis of loss of lysosomal acidification

To detect loss of lysosomal acidification, cells were stained with the azurophilic dye acridine orange (Sigma-Aldrich). At designated time-points, cells were washed three times with PBS before being incubated at 37°C in RPMI containing 5 µM acridine orange for 15 min. The cells were then washed and re-suspended in ice cold PBS and analyzed by flow cytometry.

### Cathepsin D localization and activation

To visualize cathepsin D, macrophages were loaded with 1 µM pepstatin A-BODIPY FL conjugate (Invitrogen) in complete media, for 30 min at 37°C. Pepstatin A-BODIPY FL binds cathepsin D at acidic pH [30]. After staining, cells were washed in PBS and incubated at 37°C for a further 1 h in complete media. Live cells were imaged on a Leica AF6000LX inverted microscope with a DFC 350FX RZ camera and LAS AF Lite software version 1.8, at 37°C, using a 63× lens, numerical aperture 0.7. Cathepsin D activity was measured using a fluorometric cathepsin D activity assay kit (Abcam) in accordance with the manufacturer's instructions. Fluorescence was measured on a Packard Bioscience Fusion microplate analyzer. Cathepsin D activity in each sample was expressed as percentage of a comparative sample that had been treated with 500 µM pepstatin A to act as a negative control.

### Ubiquitin pull-down assay

Ubiquitinated proteins were isolated using an enrichment kit for ubiquitin (Pierce Scientific) according to the manufacturer's instructions. Levels of ubiquitin were analyzed by Western blot, probing for ubiquitin and other proteins of interest.

### Caspase activity assay

Cellular caspase activity was measured using the Caspase-Glo 3/7 Assay (Promega) according to the manufacturer's instructions. Luminescence was measured on a Packard Bioscience Fusion microplate analyzer.

### Measurement of cytosolic pH

Intracellular pH was measured using the dye SNARF-4F carboxylic acid, acetoxymethyl ester acetate (Carboxy-SNARF-4F-AM) (Molecular Probes). 0.5×10^6^ cells per sample were pelleted and re-suspended in HEPES-buffered medium containing 10 µM carboxy-SNARF-4F-AM, and incubated at 37°C for 30 min. After incubation, cells were washed and re-suspended in fresh medium. The cells were then analyzed by flow cytometry, with the intracellular pH values being determined by measuring the ratio of fluorescent emissions at 575 nm and 635 nm. A standard curve was generated by calibration of the fluorescence ratio in buffers of different ionic strength, containing the proton ionophore nigericin, to convert this fluorescence ratio to intracellular pH.

### Detection of apoptosis

To detect loss of Δψ_m_, at the required time-points, cells were stained with 10 µM 5,5′, 6,6′-tetrachloro-1, 1′, 3,3′ tetraethylbenzimidazolocarbocyanine iodide (JC-1; Sigma-Aldrich) and analyzed by flow cytometry. Loss of Δψ_m_ was demonstrated by a loss of fluorescence on the FL-2 channel as previously described [12]. Nuclear fragmentation was detected by 4′6′-diamidino-2-phenylindole (DAPI, Molecular Probes) staining as described previously. Briefly, three hundred cells per coverslip, from at least two fields of view from the edge were counted in duplicate samples by blinded reviewers [8].

### In-vitro killing assay

Assessment of intracellular killing was carried out at designated times as before [48]. For assessment of bacterial killing, cells were infected and at 4 h washed three times in PBS before being incubated for 30 min at 37°C in RPMI containing 40 Mu penicillin and 20 µg/ml gentamicin, washed and incubated with 0.75 µg/ml vancomycin (Sigma) to kill extracellular bacteria. At the designated time cells were then washed three times in PBS before being lysed in 250 µl 2% saponin for 12 min. The lysate was made to 1 ml in PBS, and a viable count performed. Wells were lysed in triplicate for each time point.

### Hemolytic assay

Strains of bacteria were diluted to give equivalent OD600 before being pelleted, resuspended in PBS, and lysed by sonication. Red blood cells were isolated by centrifugation from defribrinated sheep blood (TCS Biosciences), washed three times in PBS and resuspended, to give a 2% solution. 50 µl of this solution was added to 50 µl PBS and placed in a round-bottomed plate. 50 µl of bacterial lysate was added per well, and the plate incubated at 37°C for 1 h. Another 50 µl PBS was added, and the plate centrifuged at 1000×g. Supernatants were analysed for the release of hemaglobin by measuring the OD at 490 nm.

### Bone marrow transfer and in vivo infection

Recipient mice were 6 week old C57BL/6J female mice (Charles River), maintained on acidified water in individual ventilated cages and irradiated with 2 doses of 550 rads separated by 4 h. Donor bone marrow, was obtained from cathepsin D deficient mice, or wild-type littermates, that had been backcrossed for 10 generations onto a C57BL/6J background. Bone marrow was isolated as described previously [39] and resuspended in HBSS at approximately 1×10^7^ cells/ml. 4 h after the second dose of radiation, 200 µl of the bone marrow cell suspension was injected into each recipient mouse via the tail vein. Bone marrow transplantation was confirmed by reconstitution of neutrophil numbers in the peripheral blood and by demonstrating alveolar macrophage expression, or absence of expression, of cathepsin D, as appropriate depending on the donor's genotype. The mice were maintained in individual ventilated cages with free access to autoclaved food and acidified water for 3 months before intratracheal instillation with 1×10^3^ or 1×10^4^ colony forming units of murine passaged type 1 *S. pneumoniae* as described previously [8]. Mice were killed 14–24 h after infection, and bronchoalveolar lavage and lungs collected. Bacterial numbers in the lung and alveolar macrophage apoptosis were assessed as previously described [8]. Neutrophil recruitment was assessed by hemocytometer counts and analysis of cytospin preparations [8]. To deplete neutrophils mice were injected ip with 100 µg anti-Ly-6G antibody (clone RB6-8C5, eBioscience) or 100 µg rat IgG (eBioscience) 24 hours prior to infection with *S. pneumoniae* [45]. This resulted in a mean reduction in neutrophil numbers in BAL of 71.4%. All experiments were performed in accordance with the UK Animals Act, authorised under a UK Home Office Licence, and approved by the animal project review committee of the University of Sheffield.

### Statistical analysis

Pooled data are expressed as mean and SEM. The indicated statistical tests were performed using Prism 4.0 software (Graphpad Inc). Significance was defined as P<0.05.

## Supporting Information

Figure S1 The number of bacteria internalized is equivalent in differentiated THP-1 cells, when exposed to pneumolysin sufficient or deficient strains of pneumococci. Differentiated THP-1 cells were infected with the designated strain of Spn (D39 or a D39 mutant which lacks pneumolysin PLYSTOP). At 4 h post-infection internalized bacteria were stained and counted, (n = 3). Internalization of D39 was also measured in cells that had been treated with pepstatin A (+PepA) as in materials and methods.
(0.26 MB TIF)

Figure S2 Phagolysosome isolation. Differentiated THP-1 cells were infected with D39. Six hours post-infection the phagolysosomes were purified on a discontinuous sucrose gradient by ultracentrifugation. Fractions from throughout the gradient were taken and separated by SDS-PAGE before being probed for pneumolysin and for the lysosomal markers pneumolysin, cathepsin D, rab5, rab7 or LAMP1. The Golgi protein GM130 was also probed for as a control for organelle contamination.
(2.28 MB TIF)

Figure S3 Validation that a pneumolysin complementation mutant restores the phenotype of the parental strain. Acridine orange (A) and JC-1 (B) staining of differentiated THP-1 cells 16 h post-infection. Cells were mock-infected (MI) or infected with D39 pneumococci, a strain overexpressing pneumolysin (SH3), a strain deficient in pneumolysin (PLYSTOP) or a complemented strain (SH3PLYSTOP). Representative histograms from one infection and graphs summarizing loss of lysosomal acidification (LLA) and inner mitochondrial transmembrane potential (Δψ_m_) from four independent infections are shown, n = 4. (C) A hemolytic assay measuring optical density (OD) at 490 nm performed on bacterial lysates of the designated strain of pneumococci, n = 3. (D) Cathepsin D activity measured in differentiated THP-1 cell lysates 14 h post-infection. Cells were mock-infected (MI), or infected with the designated strain of pneumococci in the presence (+PepA) or absence of pepstatin A, n = 3. In all graphs * = p<0.05, ** = p<0.01, one-way ANOVA with Tukey's post-test.
(1.16 MB TIF)

Figure S4 Cathepsin D, but not other cathepsins, plays a role in apoptosis induction. (A) Differentiated THP-1 cells were mock-infected (Spn-) or infected with D39 pneumococci (Spn+) infected in the presence (+) or absence (−) of inhibitors to cathepsin D (Pepstatin A; PepA), cathepsin B (CA-074Me) or cathepsins B and L (E-64d), and stained with JC-1 at 16 h post-infection, n = 3. (B) Spn− or Spn+ differentiated THP-1 cells were infected in the presence (+) or absence (−) of diazoacetyl-DL-2-aminohexanoic acid-methyl ester (DAME), and assayed for nuclear fragmentation 20 h after infection, n = 4. For both graphs * = p<0.05, two-way ANOVA. In all cases, pooled data are expressed as mean +/− SEM.
(0.50 MB TIF)

Figure S5 Reactive oxygen and nitrogen species contribute to macrophage apoptosis. Mock-infected (Spn−) or D39 infected (Spn+) differentiated THP-1 cells were incubated in the presence of (+) or absence of (−) pepstatin A, the specific iNOS inhibitor 1400W, or the antioxidant trolox, either individually, or in combination. Cells were fixed and analyzed for nuclear fragmentation 20 h post-infection, n = 4, * = p<0.05, ** = p<0.01, *** = p<0.001, one-way ANOVA with Tukey's post-test.
(0.22 MB TIF)

Figure S6 Results in monocyte-derived macrophages (MDM) replicate those seen in differentiated THP-1 cells. (A) A Western blot for the active (mature heavy chain) form of cathepsin D (Cat D) performed with whole cell lysates from mock-infected (Spn−) or D39 infected (Spn+) 16 h post-infection. The blot is representative of three independent infections in three separate donors. Acridine orange (B) and JC-1 staining (C) measuring loss of lysosomal acidification (LLA) and inner mitochondrial transmembrane potential (Δψ_m_) respectively, at 16 h post-infection, in cells mock-infected (Spn−) or infected with D39 (Spn +) in the presence (+) or absence (−) of pepstatin A (PepA), n = 4. (D) Nuclear fragmentation analysed 20 h post-infection, in Spn− or Spn+ MDM in the presence (+) or absence (−) of pepstatin A (PepA), n = 3. In all graphs * = p<0.05, one-way ANOVA with Tukey's post-test.
(0.56 MB TIF)

Figure S7 Cathepsin D deficient BMDMs exhibit similar lysosomal density and phagocytic function to wild-type (WT) BMDMs. (A) Immunohistochemistry was performed on WT and cathepsin D knockout BMDMs stained with the lysosomal marker LAMP1 (green). Slides were counterstained with DAPI (blue) to show nuclear localization. (B) WT and cathepsin D knock-out (KO) BMDMs were infected with FITC-labelled opsonized pneumococci. At 4 h post-infection the number of internalized bacteria was determined, (n = 3).
(2.11 MB TIF)

Figure S8 Cathepsin D contributes to bacterial killing through the initiation of apoptosis. (A) Differentiated THP-1 cells were mock-infected (Spn−) or exposed to D39 pneumococci (Spn+), in the presence (+) or absence (−) of zVADfmk (zVAD), zFAfmk (zFA) or pepstatin A (PepA). (A) Nuclear fragmentation in Spn− or Spn+ differentiated THP-1 cells 20 h post-infection, n = 3. (B) Cathepsin D activity measured in Spn− or Spn+ differentiated THP-1 cell lysates 14 h post-infection, n = 3. (C) Intracellular colony forming units (CFU) in Spn+ differentiated THP-1 cell lysates 16 h post-infection, in the presence or absence of the indicated inhibitors, n = 3. In all graphs ns  =  not significant, * = p<0.05, one-way ANOVA with Tukey's post-test.
(0.36 MB TIF)

Figure S9 Mice reconstituted with cathepsin D deficient bone marrow have similar numbers of alveolar macrophages to those reconstituted with wild-type bone marrow. Irradiated mice were reconstituted with either wild type (WT) or cathepsin D knock-out (CatD KO) bone marrow. The number of macrophages present in the bronchial alveolar fluid was determined by cytospin counts three months after transplantation, n = 6. There were no significant differences between groups. In all cases, pooled data are expressed as mean +/− SEM.
(0.18 MB TIF)

Figure S10 A model for the role of cathepsin D in macrophage apoptosis. After internalization of S. pneumoniae into the phagolysosome, pneumolysin (PLY) activates cathepsin D (Cat D). Lysosomal membrane permeabilization results in translocation of Cat D into an acidified cytosol. Cat D activity facilitates the binding of Mule to Mcl-1 and thus an increase in proteasomal degradation of Mcl-1 via ubiquitination. This leads to an increase in the turn-over of Mcl-1, which allows Bax and Bak activation, mitochondria outer membrane permeabilization and the initiation of downstream apoptotic features.
(1.64 MB TIF)
